# 

**Published:** 1957-01

**Authors:** 


					www 7/
?/
THE
MEDICAL JOURNAL
OF THE
SOUTH-WEST
Incorporating
The Bristol Medico-Chirurgical Journal
January 1957
VOL. 72 (i). No. 263 Price 4/-

				

